# Contribution of Segments to Overall Result in Elite Triathletes: Sprint Distance

**DOI:** 10.3390/ijerph18168422

**Published:** 2021-08-10

**Authors:** Javier Olaya, José Fernández-Sáez, Ove Østerlie, Alberto Ferriz-Valero

**Affiliations:** 1 Faculty of Health Sciences, Isabel I University, 09003 Burgos, Spain; javier.olaya@ui1.es; 2 Terres de l’Ebre Research Support Unit, Jordi Gol i Gurina University Institute for Primary Health Care Research (IDIAPJGol), 43500 Tarragona, Spain; jfernandez@idiapjgol.info; 3 Research Unit, Terres de l’Ebre Territorial Management, Catalan Institute of Health, 43500 Tarragona, Spain; 4 Department of Teacher Education, Faculty of Social and Educational Science, NTNU—Norwegian University of Science and Technology, ILU, Postboks 8900, NO-7491 Trondheim, Norway; 5 Department of General and Specific Didactics, University of Alicante, 03690 Alicante, Spain; alberto.ferriz@ua.es

**Keywords:** race, performance, triathlon, swimming, cycling, running

## Abstract

As an alternative to analysing the contribution of performance in specific segments of a triathlon to the overall result as measured in terms of time or position, which has several limitations, previous studies have instead analysed the performance indicator in triathlon. Therefore, the purpose of the study was to analyse the relationship between performance in specific segments and overall performance in terms of sprint distance in elite triathletes through the triathlon performance indicator, instead of using time or position. The official sprint distance results from World Triathlon Series elite events from 2012 to 2019 were examined. In total, 2144 entries were considered, 1143 of which were men and 1001 were women. Performance in the cycling segment presents the best concordance with the overall performance for both elite men (ICC_a_ = 0.871, IC95% = (0.711–0.927)) and elite women (ICC_a_ = 0.907, IC95% = (0.875–0.929)). Although the performance in the running segment does not show the best concordance with the overall performance, the position in this segment does better explain the overall position, especially in elite men and in draft-legal races. These results can support coaches and athletes to identify a specific profile of the strengths and weaknesses of triathletes in competitions, in comparison to their rivals, over a specific distance.

## 1. Introduction

The triathlon is an endurance combination sport that comprises a sequential swim, swim-to-cycle transition (T1), cycle, cycle-to-run transition (T2), and run over a variety of “long” or “short” distances [1]. The International Triathlon Union (ITU) stipulates the standard races distances as sprints (750 m swim, 20 km cycle and 5 km run) and Olympic distance (1.5 km swim, 40 km cycle and 10 km run) events. In 2009, the ITU changed the racing format in a series of Olympic events called the World Triathlon Series (WTS), which replaced the single World Championship. Specifically, in 2012, one of the main features of the calendar was the inclusion of the sprint distance races in the WTS and World Cups, which, until then, were held using the Olympic distance standards. Currently, the sprint distance is the most performed distance in lower categories such as senior and junior [2]. Competition times for each distance are completely different, ranging from 50 to 70 min for the sprint distance to several hours for the Olympic and long distances [3]. Therefore, the physiological training objectives for athletes are different for the Olympic distance [4] and the sprint distance [3,5]. The study of the contribution of segments and transitions to the overall results of the competition has been widely focused on the Olympic distance [6,7,8,9,10,11,12]. However, very little data are available on sprint distance triathlon performance, especially for high-level international races [13], despite the fact that, depending on the distance of the triathlon, the discipline for predicting overall triathlon performance changes [14].

Using the absolute time as a comparative variable of reference between different triathlons is difficult because each triathlon takes place in specific meteorological conditions, with varying distances and circuits, or with triathletes taking different tactical positions [15]. For example, the elite male winner of the WTS sprint distance in the Stockholm 2012 event won, in that year, with an overall time of 54:24, while in 2014 that same athlete won again, but with an overall time of 57:31. In consequence, all of these factors may affect the overall finishing time [16]. In addition, the analysis of the contributions of segments to the overall result, measured in terms of time, presents some limitations since the duration of the segments are not the same and the contribution to overall time will be affected by the longer or shorter duration of each segment [17]. Nevertheless, many studies [3,6,9,18,19] have analysed the contributions of the time of each segment to the overall result, reaching different conclusions. In sprint racing, Horne [18] points out that in the World Championship, with athletes divided according to age group, cycling had the strongest relationship with finishing time, while the lowest correlation with finishing time was found in the swimming section, although this study was carried out in draft-illegal races. Likewise, Sousa et al. [14] claimed that the best predictor for Sprint distance is cycling. Regarding the running segment, these same authors concluded that the importance of running in the prediction of overall performance diminishes with decreasing race distance. By contrast, it has also been concluded that the running segment should be the primary determinant of success in high-level short distance triathlon races; however, it should be noted that this investigation was carried out by physical education student volunteers instead of elite triathletes [3]. Nevertheless, similar results were observed in the Olympic distance [14]. Figueiredo et al. [6] highlighted that strategies to improve in running segment should be the main focus in the preparation of the Olympic distance. Because transitions can be important to overall performance, especially in shorter races, it is worth noting the lack of investigations on these segments for sprint distance [14] compared to Olympic distance events [9,16,19]. For Olympic distance events, Cejuela et al. [19] found a low correlation between T1 and the overall result, although the lost time in this transition was different for each swimming pack. Other authors [9] also noted the tactical importance of entering T2 at the front of the group to avoid collisions or jams in Olympic distance events.

The position of the segments and the influence on overall classification were analysed in other research studies [7]. However, once again, the determination of performance—this time by position—presented some limitations. For example, regarding the previous example of the WTS in the Stockholm 2012 event, the third-place athlete (54:35 overall time) took the podium by beating the fourth-place athlete (54:36 overall time) by 1 s. By position, it is not possible to appreciate greater or lower differences in time between athletes, who are logically assumed to have shown greater or worse relative performance, as the position is an ordinal number [17]. Either way, to date, the analysis of performance through time and position has been widely studied in triathlons despite these limitations.

For this reason, as an alternative, previous research has proposed a triathlon performance indicator (PI) created mainly to analyse young triathletes’ performance and identify performance factors to develop triathlon talent [20]. Thus, the purpose of the study was to analyse the relationship of the performance of each segment with the overall performance of elite triathletes in sprint distance events through the triathlon performance indicator, instead of time or position.

Finally, it was initially hypothesized that:

**Hypothesis** **(H1).** *The performance indicator in the swimming segment has the lowest agreement with the overall performance in the sprint triathlon race.*

**Hypothesis** **(H2).** *The performance indicator in the cycling segment has the best agreement with the overall performance in the sprint triathlon race.*

**Hypothesis** **(H3).** *The performance indicator in the running segment has the lowest agreement with the overall performance in the sprint triathlon race.*

**Hypothesis** **(H4).** *The performance indicator in the transition segments has no agreement with the overall performance in the sprint triathlon race.*

## 2. Material and Methods

A correlational study design was used because this research concerned international elite-level competitions. Thus, the times of the swimming, cycling and running segments, the times taken to complete transitions, and the overall times of the races were analysed. Therefore, the research design was based on an observational model without interference in the natural context of the events under study.

### 2.1. Participants

All data originated from the official results of the WTS elite men’s and elite women’s sprint distance events from 2012 (the first year in which sprint distance events were included in the WTS) to 2019. A total of 2144 entries were examined, 1143 of which were elite men and 1001 were elite women. All races that included no information on swimming, cycling and running segment times, T1 and T2 and overall time were excluded. Races in which the sprint distance was altered due to technical or environmental issues were also excluded.

### 2.2. Procedures

All segment times of the races included in the WTS were recorded through a chip-based timing system that could obtain highly accurate records of individual performance according to the portions of the race. This timing system was the system used by the International Triathlon Union. Therefore, it was possible to use the performance indicator in triathlons as a dependent variable to analyse performance in elite men and elite women triathletes. The variable is expressed from 0 to 10,000 where 10,000 is the best segment time, and thus, the best performance. The formula multiplies the result by 10,000 to give more accuracy to the comparison of results. Thus, the formula can differentiate a single second between runners’ times in, e.g., Ironman distance events where the winning time is around 8 h.

$$\mathrm{OPI}=\frac{\mathrm{Winner}\;\mathrm{time}}{\mathrm{Personal}\;\mathrm{time}}\;\times \;10,000$$

This calculation provides a performance indicator for each segment and transition in a triathlon [17]: the swimming performance indicator (SPI), cycling performance indicator (CPI) and running performance indicator (RPI), and also for each swim-to-cycle transition (T1PI), cycling segment, and cycle-to-run transition (T2PI), as well as for the overall performance indicator (OPI).

### 2.3. Statistical Analysis

To analyse the concordance of the performance of each segment with the overall performance of the competition, the absolute agreement intraclass correlation coefficient (ICC_a_) was calculated, which considers any difference between performances as a discordance. The consistency intraclass correlation coefficient (ICC_c_), on the other hand, does not consider the constant differences between performances. The ICC_a_ and ICC_c_ take values between 0 and 1, in which the maximum possible agreement corresponds to a value of ICC = 1. In this case, all observed variability would be explained by differences between subjects and not by differences between measurement methods. ICC = 0 is obtained when the observed concordance is the same as the difference that would be expected to occur only by chance. To interpret the magnitude of concordances between measurement variables, the following criteria were adopted: <0.1 (trivial), 0.1–0.3 (small), 0.3–0.5 (moderate), 0.5–0.7 (large), 0.7–0.9 (very large) and 0.9–1.0 (almost perfect) [21].

The same criteria were adopted to interpret the Spearman range correlation coefficient used to analyse the degree of association between the performance indicator of each segment and the overall performance indicator.

The Bland-Altman method is a graphical method that allows the comparison of two measurement techniques on the same quantitative variable; in this case, the comparison between the performance indicators of the different segments with the overall performance indicator of the competition. To assess the degree of agreement between the performance indicators of each segment and the overall performance indicator, the method of graphical representation proposed by Bland and Altman was used [22,23] for each segment, using the average values of the performance indicators against their differences. The average of the differences in the values corresponds to the systematic error that quantifies how much the performance of each segment overestimates or underestimates the overall performance [24]. In addition, the precision with which the performance indicator of each segment estimates the overall performance, which represents the degree to which the values are grouped around the average, quantified through the interval of ±1.96 standard deviations of the differences between the two measurement systems. All data were analysed statistically with the software Statistical Package for The Social Sciences (v.24.0 SPSS Inc., Chicago, IL, USA) and using a Microsoft Excel spreadsheet. Significance was accepted at *p* < 0.05.

## 3. Results

Table 1 shows the relationship between the performance indicators of the segments and the overall performance indicator for elite men and elite women. Large, very large and almost perfect correlations were found between the performance results of the three segments and the overall performance indicator in both sexes. Performance in the cycling segment presents the best agreement with the overall performance for both elite men (ICC_a_ = 0.871, IC95% = (0.711–0.927)) and elite women (ICC_a_ = 0.907, IC95% = (0.875–0.929)). Furthermore, the best concordance with overall performance was found in the cycling segment for both elite men (ICC_c_ = 0.902, IC95% = (0.890–0.913)) and elite women (ICC_c_ = 0.916, IC95% = (0.905–0.925)). Significant agreement was also found between the running segment and overall performance for both elite men (ICC_a_ = 0.564, IC95% = (0.209–0.815)) and elite women (ICC_a_ = 0.563, IC95% = (0.174–0.805)). In addition, significant agreement with overall performance was found in the swimming segment in both elite men (ICC_a_ = 0.514, IC95% = (0.346–0.628)) and elite women (ICC_a_ = 0.586, IC95% = (0.511–0.647)). Performance in transitions showed little agreement and poor correlation to overall competition performance in elite men and elite women. Concerning the analysis of the position, in elite men, classification according to running performance indicator best explains the overall classification (ρ = 0.810, *p* < 0.001).

**Figure 1:**
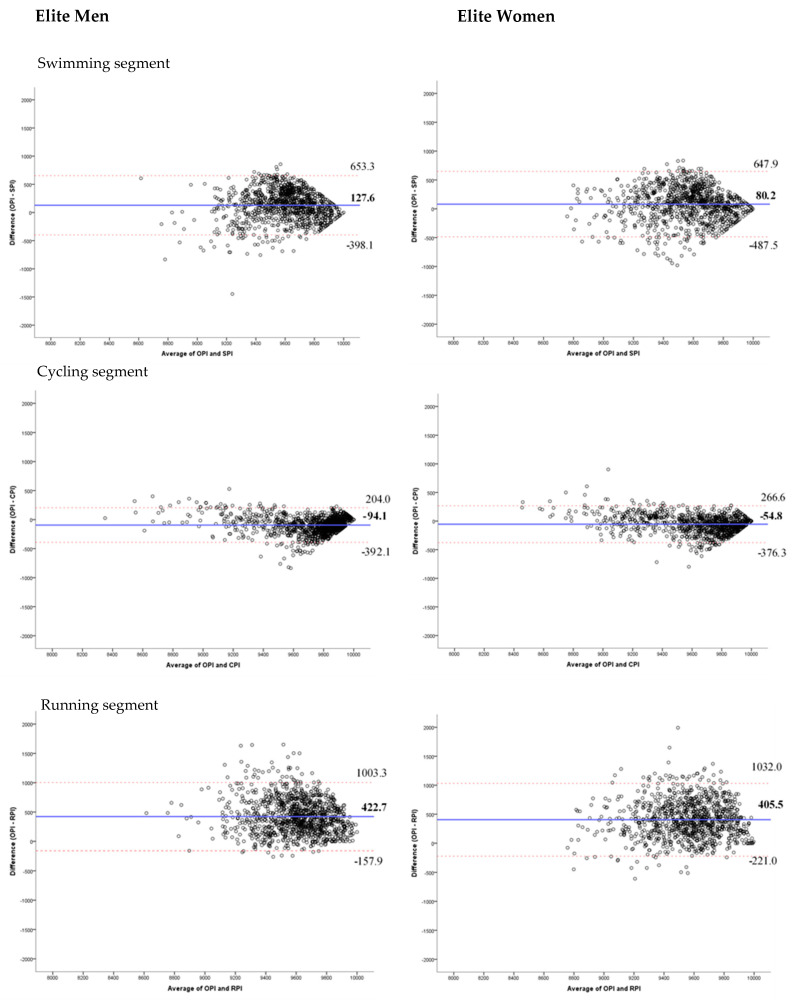
Bland-Altman plots showing differences in performance according to the swimming performance indicator (SPI), cycling performance indicator (CPI), running performance indicator (RPI) and overall performance indicator (OPI). The red lines represent the upper and lower 95% limits of agreement, whereas the blue line represents the bias.

**Figure 2:**
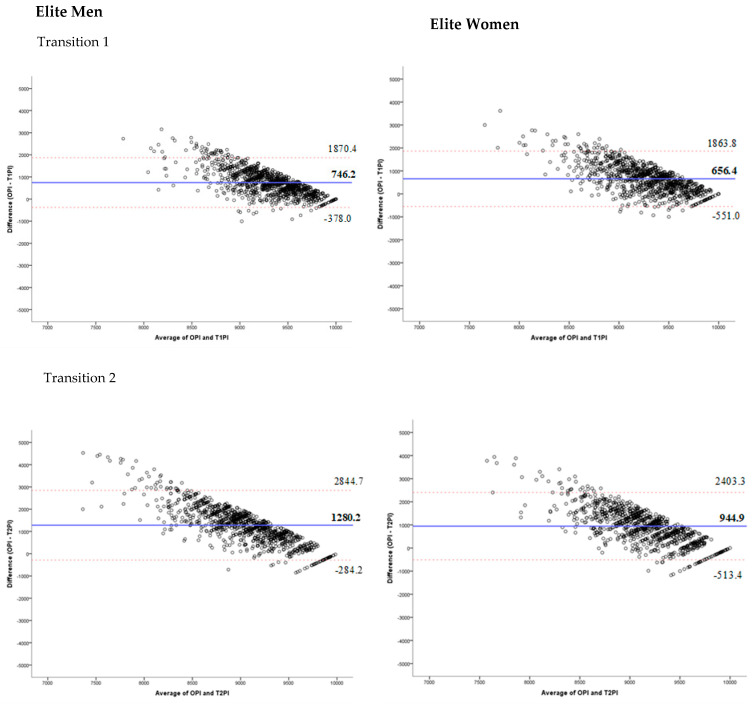
Bland-Altman plots showing differences in performance according to the transition 1 performance indicator (T1PI), transition 2 performance indicator (T2PI) and overall performance indicator (OPI). The red lines represent the upper and lower 95% limits of agreement, whereas the blue line represents the bias.

Bland-Altman plots present the difference of the averages between the overall performance and performance in specific segments (Figure 1) and performance during transitions (Figure 2). Figure 1 shows that the average difference between overall performance and swimming performance is 126.6 in elite men and 80.17 in elite women. In contrast, cycling performance is higher than overall performance, specifically 94.05 in elite men and 54.81 in elite women. The biggest difference was found between overall and running performance, which ranges from 422.73 in elite men to 405.47 in elite women. In all three segments and in both sexes, the concordance between performance in specific segments and overall performance increases for performance rated above 9500. The trivial concordance between performance during transitions and overall performance is shown in Figure 2.

## 4. Discussion

The performance indicator scores each athlete according to the difference between their completion time and that of the athlete who attained first place in the race. In this sense, we have an indicator that scores each athlete specifically for each category (elite men and elite women) and race, allowing us to compare performances between races, assuming some limitations. For example, the measuring of performance according to the winning time is limited in that it depends on the presence of talented triathletes in any competition, although this study deals with a wide range of professionals in the highest competition (WTS). However, the winning time provides results that are more suitable to assess high performance than average or standardized times. This was the first study to investigate the contribution of the segments to overall performance in sprint distance for elite men and elite women using a significantly different measure than absolute time or position, which are the most common performance indicators in triathlons.

The results of the present investigation obtained in the different segments and transitions are explained below. Firstly, the swimming performance indicator (SPI) shows a large agreement with the overall performance indicator (OPI) in both elite men (ICC_a_ = 0.514, IC95% = (0.346–0.628)) and elite women (ICC_a_ = 0.586, IC95% = (0.511–0.647)), while only a moderate correlation is found between swimming position and overall position, again in both elite men (ρ = 0.370, *p* < 0.001) and elite women (ρ = 0.397, *p* < 0.001). Although, in elite women, the SPI does not show the least absolute agreement between segment performance and OPI, this segment is the one that reflects the least concordance regarding the OPI in both elite men (ICC_c_ = 0.565, IC95% = (0.511–0.612)) and elite women (ICC_c_ = 0.604, IC95% = (0.553–0.649)). In this case, the results of the present study agree with those of Horne [18], who revealed that, in the sprint distance World Championship event, divided by age-group, the time of the swimming section showed the lowest correlation with the overall finishing time. Similar results were found in Olympic distance events, where Cejuela et al. [19] revealed that the swimming discipline was the segment of the race that had the lowest correlation with overall placement. Therefore, the first Hypothesis (H1) was supported.

Secondly, the main result of this research is that performance in the cycling segment (CPI) is the most correlated with the OPI in both sexes. Specifically, very large and almost perfect agreements were found between CPI and OPI in elite men (ICC_a_ = 0.871, IC95% = (0.711–0.927)) and elite women (ICC_a_ = 0.907, IC95% = (0.875–0.929)), respectively. These results agree with those proposed by Horne [18], Cejuela et al. [19] and Sousa et al. [14], in which the cycling segment had the strongest relationship with overall finishing time, with no differences found between sexes. Therefore, the second Hypothesis (H2) was supported.

From a practical point of view, swimmers who do not finish the swimming segment in the leader group (chasing group) but manage to link in the bike segment with the first pack (lead group) will have better performance in the cycling segment.

Thirdly, performance in the running segment (RPI) also showed large agreement with OPI, with similar values being observed in both sexes according to Cejuela et al. [19]. Therefore, the third Hypothesis (H3) was not supported. Nevertheless, it is worth noting the very large correlations between the position of the running segment and the overall position. Specifically, in elite men, the position in running segment is the one that best explains the overall classification (ρ = 0.810, *p* < 0.001), even better than cycling position (ρ = 0.663, *p* < 0.001). This is logically consistent because in sprint distance events, athletes draft while swimming as the depression made in the water by a leading swimmer decreases the passive drag of the following swimmers by 10 to 26% [1]. Similarly, cycling in a sheltered position makes it possible to reduce expiratory flow, oxygen uptake, heart rate and blood lactate concentrations in contrast to biking alone [25]. Therefore, in races where drafting is allowed, athletes usually draft while swimming and cycle in different packs that are very close together, until they start to dispute the race in the running segment [25,26]. In consequence, in similar races, for instance, in Olympic distance events, previous studies highlight that the strategies to improve the time in the running segment should be the main focus in the preparation for short distance triathlons [6,18]. It is perhaps for this reason that, considering the three segments, the widest range of IC95% is recorded in the running segment in both elite men (0.209–0.815) and elite women (0.174–0.805). This analysis of the contribution of performance and segment positions to overall performance and ranking could allow for the identification of a specific profile of the strengths and weaknesses of triathletes in competitions in comparison to their rivals over a specific distance. In addition, it is recommended that the triathlete perform sport-specific testing to assess training zones for cycling and running [27].

Fourthly, transitions have also been studied in the different distances of the triathlon competitions. The results of this study show that performance in transitions showed little concordance and poor correlation to overall competition performance in men and women. As the triathlon is a combined and endurance sport, any analysis of performance over time will give more importance to segments than to transitions because they account for a small percentage of the race time. Indeed, some authors have calculated the sums of the times taken in transitions to cycling and running; however, transitions can have independent influence on overall performance [14]. For example, in Olympic distance, the variations in transitions represent <0.1% of the total time [28]. These findings are in agreement with previous studies on Olympic distance events. Cejuela et al. [19] found a low correlation between T1 and overall results; however, the lost time in T1 was different for each swimming pack (when 5 sec. gaps between swimmers were taken to indicate a different pack). Piacentini et al. [16] concluded that quicker exits from T2 (and hence, lower time loss) will certainly be beneficial for overall performance and final positioning [16]. Thus, the position reached by the transition seems to be more important in tactical and qualitative terms than in quantitative ones. Therefore, the fourth Hypothesis (H4) was supported.

Regarding sex differences, some authors suggest that future studies are required to clarify why the sex difference in running is greater compared to swimming and cycling in international short distance triathlon races with drafting [10]. In the current literature, some authors point out that men were shown to be faster triathletes than women [12], while other analyses did not find any significant effect of sex on the contribution of each discipline (%) to overall performance in all four triathlon distances [14]. Barbosa et al. [29] suggested that cycling was the discipline with the highest influence on overall race time for both sexes in long-distance events. Similarly, the main results of the present study also show that performance in the cycling segment (CPI) is the most correlated with the overall performance indicator (OPI) in both sexes, confirming these results also in short-distance events. However, men and women never compete together; therefore, the times of both sexes could never be compared to interpret the performance of elite men and elite women. Therefore, the performance of the two sexes should be compared specifically for each category; for example, by comparing the performance of the other athletes with the first athlete of the race, as a performance indicator.

## 5. Conclusions

The purpose of the study was to analyse the relationship of the performance of each segment with the overall performance of elite triathletes in sprint distance events through the triathlon performance indicator. Generally, most of the correlations between the segment performance indicator and the overall performance indicator are greater than the correlations of the segment position and the overall position. Either way, it would be preferable to use these two variables to achieve a more complete analysis of the race. Performance in the cycling segment shows the best correlation with overall performance in sprint distance events in both elite men and elite women. For this reason, triathletes and coaches should focus their attention on training that is specific to this segment. Large correlations also were found between running performance and overall performance. Although the performance in the running segment does not show the best correlation with the overall performance, the position in this segment does explain the overall position, especially in elite men and in draft-legal races such as the ones included in this study. In addition, large correlations between swimming performance and overall performance were found in both sexes. By making use of the performance indicator, this analysis allows coaches to identify specific profiles of the strengths and weaknesses of triathletes in competitions, and to draw comparisons with their rivals over specific distances. These results are useful for triathlon coaches because they provide information on sprint distance races that have, up to now, been insufficiently studied since they were not included in the World Championship until 2012. Future studies should examine the performance indicator over a wider range of distances and in lower category events.

## Figures and Tables

**Table 1:** Intraclass correlation coefficient of absolute agreement and concordance, and Spearman range correlation coefficient between the overall performance indicator and the three segments in the years 2012–2019.

	ICC_a_ (IC95%)m ^b^	*p*	ICC_c_ (IC95%)	*p*	dρ	*p*
**Elite Men**						
SPI	0.514 (0.346–0.628)	<0.001	0.565 (0.511–0.612)	<0.001	0.370	<0.001
T1PI	0.130 (0.088–0.311)	<0.001	0.287 (0.200–0.365)	<0.001	0.255	<0.001
CPI	0.871 (0.711–0.927)	<0.001	0.902 (0.890–0.913)	<0.001	0.663	<0.001
T2PI	0.043 (0.001–0.130)	0.006	0.138 (0.032–0.232)	0.006	0.137	<0.001
RPI	0.564 (0.209–0.815)	<0.0001	0.797 (0.772–0.819)	<0.001	0.810	<0.001
**Elite Women**						
SPI	0.586 (0.511–0.647)	<0.001	0.604 (0.553–0.649)	<0.001	0.397	<0.001
T1PI	0.148 (0.067–0.319)	<0.001	0.270 (0.176–0.354)	<0.001	0.219	<0.001
CPI	0.907 (0.875–0.929)	<0.001	0.916 (0.905–0.925)	<0.001	0.773	<0.001
T2PI	0.032 (−0.039–0.039)	0.094	0.080 (−0.042–0.187)	0.094	0.074	0.019
RPI	0.563 (0.174–0.805)	<0.001	0.711 (0.741–0.797)	<0.001	0.718	<0.001

SPI: Swimming Performance Indicator. T1PI: Transition 1 Performance Indicator. CPI: Cycling Performance Indicator. T2PI: Transition 2 Performance Indicator. RPI: Running Performance Indicator. OPI: Overall Performance Indicator. ^a^ Absolute Agreement Intraclass Correlation Coefficient. ^b^ 95% Confidence Intervals. ^c^ Concordance Intraclass Correlation Coefficient. ^d^ Spearman range Correlation Coefficient.

## Data Availability

All result data used in the analysis in this paper are available from the World Triathlon website: Available online: https://www.triathlon.org (accessed on 19 March 2020).
